# A Comparison of Methods for Classifying Clinical Samples Based on Proteomics Data: A Case Study for Statistical and Machine Learning Approaches

**DOI:** 10.1371/journal.pone.0024973

**Published:** 2011-09-28

**Authors:** Dayle L. Sampson, Tony J. Parker, Zee Upton, Cameron P. Hurst

**Affiliations:** 1 Tissue Repair and Regeneration Program, Institute of Health and Biomedical Innovation, Queensland University of Technology, Kelvin Grove, Queensland, Australia; 2 Workforce Health Innovation Program, Institute of Health and Biomedical Innovation, Queensland University of Technology, Kelvin Grove, Queensland, Australia; 3 Faculty of Health, School of Public Health, Queensland University of Technology, Kelvin Grove, Queensland, Australia; Dana-Farber Cancer Institute, United States of America

## Abstract

The discovery of protein variation is an important strategy in disease diagnosis within the biological sciences. The current benchmark for elucidating information from multiple biological variables is the so called “omics” disciplines of the biological sciences. Such variability is uncovered by implementation of multivariable data mining techniques which come under two primary categories, machine learning strategies and statistical based approaches. Typically proteomic studies can produce hundreds or thousands of variables, ***p***, per observation, ***n***, depending on the analytical platform or method employed to generate the data. Many classification methods are limited by an ***n***≪***p*** constraint, and as such, require pre-treatment to reduce the dimensionality prior to classification. Recently machine learning techniques have gained popularity in the field for their ability to successfully classify unknown samples. One limitation of such methods is the lack of a functional model allowing meaningful interpretation of results in terms of the features used for classification. This is a problem that might be solved using a statistical model-based approach where not only is the importance of the individual protein explicit, they are combined into a readily interpretable classification rule without relying on a black box approach. Here we incorporate statistical dimension reduction techniques Partial Least Squares (PLS) and Principal Components Analysis (PCA) followed by both statistical and machine learning classification methods, and compared them to a popular machine learning technique, Support Vector Machines (SVM). Both PLS and SVM demonstrate strong utility for proteomic classification problems.

## Introduction

Protein studies have grown tremendously over the past decade with traditional methodologies advancing from the analysis of single gene products [1] to multiplex protein assays. Proteomics is a field of research that aims to holistically assay and characterise the protein complement within an organism, sample or tissue type. The current technology allows scientists to gather information on hundreds to thousands of proteins or peptides [2], [3] simultaneously using the one platform [4]. Such methodologies lend themselves to use within biomarker discovery projects due to their high throughput capacity and the large number of simultaneously measured variables produced within a single experiment.

While proteomic evaluation has improved research output in a variety of disciplines, it has also caused a number of problems relating to interpretation and analysis of simultaneously measured variables. These problems are similar to those encountered by researchers investigating gene expression. A number of distinct properties are observed within a proteomic dataset, each of which need to be considered when deciding on an appropriate analytical technique. The most notable is the so called “curse of dimensionality” where ***n***≪***p*** [5], [6]. That is, the number of observations, ***n***, is often far smaller than the number of variables (proteins or peptides), ***p***. This can lead to a number of problems which limit the generalisability and therefore clinical utility of any resulting diagnostic tools. This is relevant to this type of study due to the capacity of proteomic research to produce hundreds and thousands of variables (usually for a limited number of observations) depending on the method and platform employed. Often the variable intensities are highly correlated, this renders analysis methods that consider the mass units separately as inappropriate, a detail which has been ignored in previous studies [7]. High correlation is a result of certain proteins being up-regulated which will then have an effect on the up/down regulation of another which may or may not be related to the covariates of interest. For example a protein may differ among males and females but is also strongly correlated with the pathological state of a disease.

The properties of proteomic datasets discussed above can hamper the development of a robust classifier which is used to distinguish between group states (e.g. discriminating between diseased and non-diseased individuals). To circumvent these difficulties a common processing step often used prior to multivariate analysis is to reduce the dimensionality of the raw data [8]. The most common approach involves filtering, for example carefully choosing a specific selection of statistically relevant variables (proteins or peptides) prior to the model development process. This approach affords removal of any redundant or extraneous variables. Often the number of biomarkers selected can be altered based on the stringency of the variable selection process which is a user-defined meta-parameter. However, care should be taken that the same data used to tune the meta-parameter should not be used for subsequent classification. Steps such as holding out data, especially for tuning avoid this issue.

Alternately, the more popular approach in biomarker discovery research is to leave all the variables in the dataset, and apply dimension reduction strategies to project the mass units to a more informative lower-dimensional space. Such efforts allow those mass units that truly influence class separation to be obvious, while the rest remain in the background. Dimension reduction also accounts for the effects of highly correlated variables, a key characteristic of proteomic and genomic data. In addition, the analyst might choose to use a combination of variable selection and dimension reduction strategies to produce an informative set of biomarkers that achieve good classification results [9]. However this decision is often influenced by the analysts' choice of classifier, of which there are numerous options [10].

A number of techniques have been used in the past for the analysis of proteomic data. These include computation methods such as support vector machines (SVM), artificial neural networks (ANN) and random forests (PLS-RF), as well as model-based approaches like Partial Least Squares-Linear Discriminant Analysis (PLS-LDA) and Principal Components Regression-Linear Discriminant Analysis (PCR-LDA).

Willingale *et. al.* (2006) used SVM, ANN, genetic algorithms and Decision Forests on data produced on a Matrix-assisted laser desorption/ionisation – Time of flight mass spectrometry platform (MALDI-TOF/MS) from heart failure patients. They built their classifiers using a training set consisting of 100 heart failure and 100 control participants, and tested it using 32 heart failure and 20 control participants. Each classifier performed well with the authors concluding that one in particular, which achieved 88.5% correct classification on the test set, will be followed up with MS/MS analysis techniques [11]. Smith *et. al.* (2007) used SVM to classify early phase response to multimodal neoadjuvant therapies used on rectal tumour patients. A SVM classifier was built using seven time points, the classifiers had a sensitivity range between 25–87.5% and a specificity range of 64–80%. A key limitation of this study, however, was the insufficient number of observations (n = 20) on which the classification rule was built on [12]. Others have also used computational classification approaches with varying degrees of success [13], [14].

Purohit and Rocke (2003) used supervised and unsupervised dimension reduction and classification techniques which initially incorporated PCA to reduce dimensionality, followed by hierarchical cluster analysis for visual classification of proteomic data between healthy and diseased patients. In addition they assessed combinations of PLS and PCR with logistic regression and discriminant analysis methods for classification, demonstrating the strength of PLS-based classification which out-performed PCR-based methods [15].

Lee *et. al.* (2003) applied SVM and an ANN algorithm to their proteomic data boasting a training set accuracy of 100% and a leave-one-out-cross-validation (LOO-CV) accuracy of 95.1% for ANN and a 100% accuracy on the training set. However, they misclassified 5 out of 41 observations using SVM. This was compared to 100% training set accuracy and 85% accuracy on a test set using a simple two component PLS-DA model [16].

Liu *et. al.* (2008) incorporated PLS based methods into an ovarian cancer classification problem [17]. They compared PLS-LDA, PLS- *k* nearest neighbour (PLS-KNN), PLS-logistic regression (PLS-LR) and PLS-ANN to a range of PCA-based classification methods. Their findings suggest that PLS dimension reduction followed by a logistic regression (LR) classification produces improved results from that of PCA-based methods and other PLS approaches.

Rajalahti *et. al.* (2009) used PLS to reduce dimensionality followed by discriminant analysis to classify between cerebrospinal fluid (CSF) samples and CSF samples spiked with peptide standards [18]. They also compared three popular variable selection methods commonly used, one of which was based on PLS weights similar to Purohit and Rocke (2003).

Here we define computational methods as those which do not produce a functional model and were developed in the machine learning literature. A statistical method refers to a method that results in an explicit classification rule that clearly relates the features to class membership, such methods originally came from the multivariate analysis literature. Computational methods are popular and are often used on proteomic data, however they can be cumbersome and don't necessarily outperform statistical methods. This was demonstrated in a recent proteomic competition [19], [20] where simple PCA-based techniques [21], [22] outperformed novel computational approaches [19]. Furthermore, the absence of a functional model makes the interpretation of results using computational methods limited, where often the only thing known based on such methods is the success of classification.

This manuscript aims to demonstrate the utility and versatility of PLS-based classification methods on clinical proteomic datasets. Here, PLS-LDA, PLS-RF, SVM and PCA-LDA classification rules have been objectively compared on a range of trimmed (undergone variable pre-selection) and untrimmed datasets. We demonstrate that SVMs produced the more efficient classifier on most of the datasets tested, although, PLS-based classifiers produced models with additional meaningful information. They yield protein loadings, lend themselves to visualization and produce (when used in conjunction with a statistical classifier) functional models. An additional aspect of PLS-based methods is the speed at which the algorithm works and the efficient nature in which they reduce the complexity of the data.

## Results

### Dimension reduction-based methods (PLS & PCA)

The misclassification rate (MCR) of both the Gaucher dataset and the OC data indicates that these two datasets responded favourably to variable pre-selection. Whereas the MCR for the LC and CRC datasets indicates they were not largely affected by variable pre-selection. We expected negligible performance differences between variable selection and no variable selection with these two datasets in particular, as they did not contain all of the original mass units, having undergone previous filtering [14]. The classification results for each of the dimension reduction methods are presented in *Table 1*.

**Table 1:** Dimension Reduction Classifier Performance Summary.

Method	MCR	AUC	Spec	Sens	No. Components	Data set
pls.lda (full)	0.287	0.635	0.694	0.75	12	Gaucher
pls.rf (full)	0.343	0.75	0.707	0.636	3	
pca.lda (full)	0.231	0.992	0.779	0.794	17	
pls.lda (trimmed)	0.115	0.823	0.859	0.918	7	
pls.rf (trimmed)	0.171	0.919	0.852	0.821	7	
pca.lda (trimmed)	0.046	0.992	0.918	0.995	6	
pls.lda.lung (full)	0.196	0.889	0.778	0.837	5	Lung cancer
pls.rf.lung (full)	0.225	0.85	0.764	0.794	6	
pca.lda.lung (full)	0.2	0.897	0.756	0.85	9	
pls.lda (trimmed)	0.199	0.881	0.79	0.819	5	
pls.rf (trimmed)	0.232	0.841	0.751	0.794	6	
pca.lda (trimmed)	0.217	0.88	0.741	0.83	17	
pls.lda.CRC (full)	0.089	0.954	0.853	0.966	5	Colorectal cancer
pls.rf.CRC (full)	0.113	0.951	0.88	0.896	8	
pca.lda.CRC (full)	0.089	0.97	0.862	0.959	10	
pls.lda (trimmed)	0.089	0.951	0.855	0.963	6	
pls.rf (trimmed)	0.119	0.941	0.89	0.877	8	
pca.lda (trimmed)	0.11	0.952	0.845	0.933	2	
pls.lda (full)	0.26	0.478	0.7	0.784	8	Ovarian cancer
pls.rf (full)	0.286	0.794	0.678	0.722	7	
pca.lda (full)	0.315	0.777	0.627	0.757	17	
pls.lda (trimmed)	0.159	0.914	0.807	0.811	3	
pls.rf (trimmed)	0.191	0.897	0.818	0.81	9	
pca.lda (trimmed)	0.157	0.931	0.787	0.892	5	

The performance summary (MCR = Misclassification rate, AUC = Area under the curve, Sens = Sensitivity, Spec = Specificity, No. Components = the number of components used in the model) of each classifier for both the full dataset (“full”) and the trimmed dataset (“trimmed”) that underwent variable selection using a univariate moderated t-statistic. These are mean values based on 1000 bootstrap samples for each dataset except the OC data which used 200 bootstrap samples.

**Figure 1:**
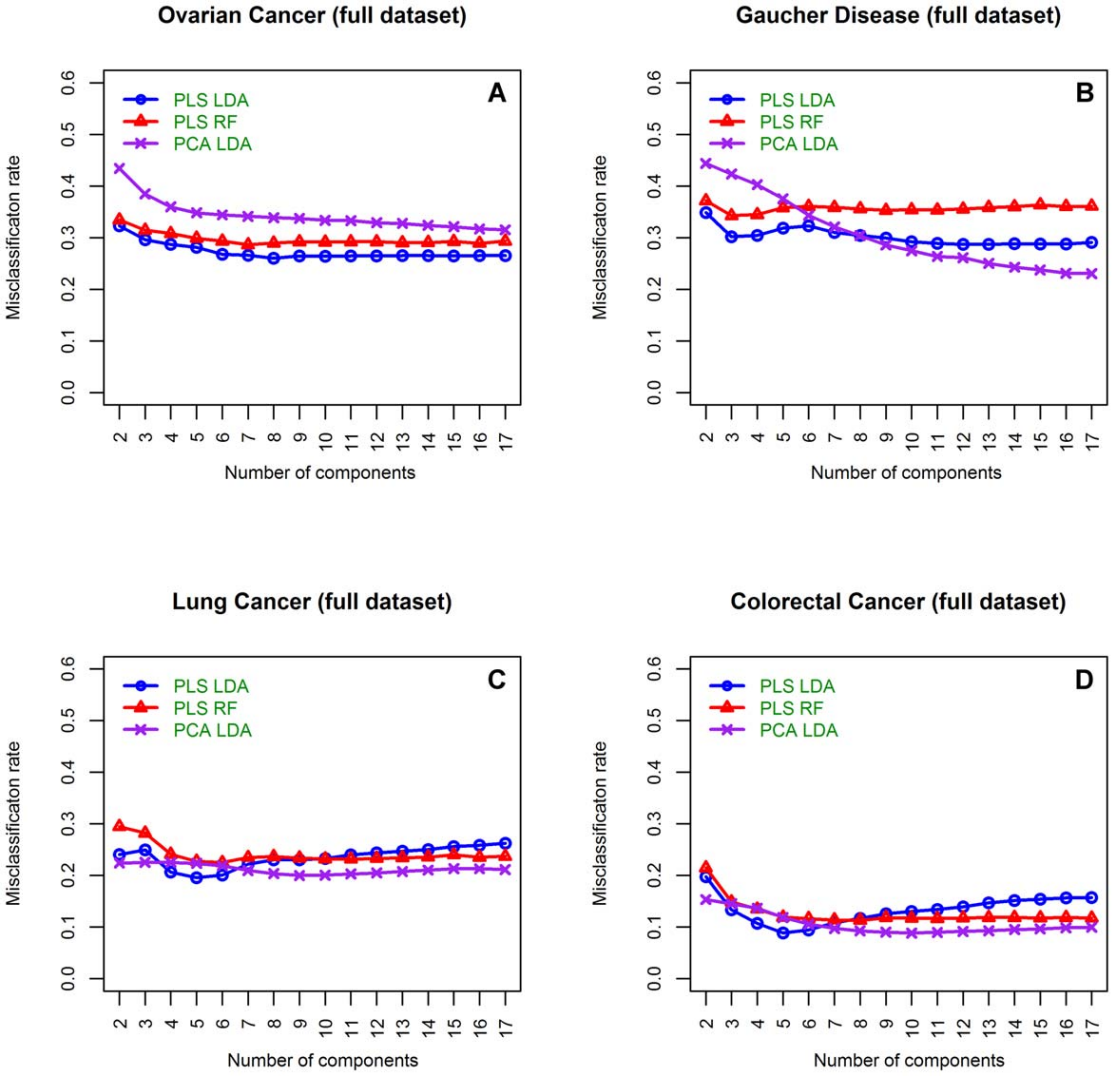
Misclassification rates of dimension reduction classifiers using the untrimmed datasets. Mean misclassification rates for each of the dimension reduction-based methods using the full dataset (all variables) in the dataset to build the classification model. **A**) Is from the OC dataset [16], **B**) is from the Gaucher disease dataset [46], **C**) is from the LC datasets and **D**) is from the CRC dataset [14]. Blue circles illustrate PLS-LDA classification results, red triangles are from a PLS-RF classifier and purple crosses show results obtained from a PCA-LDA classifier.

**Figure 2:**
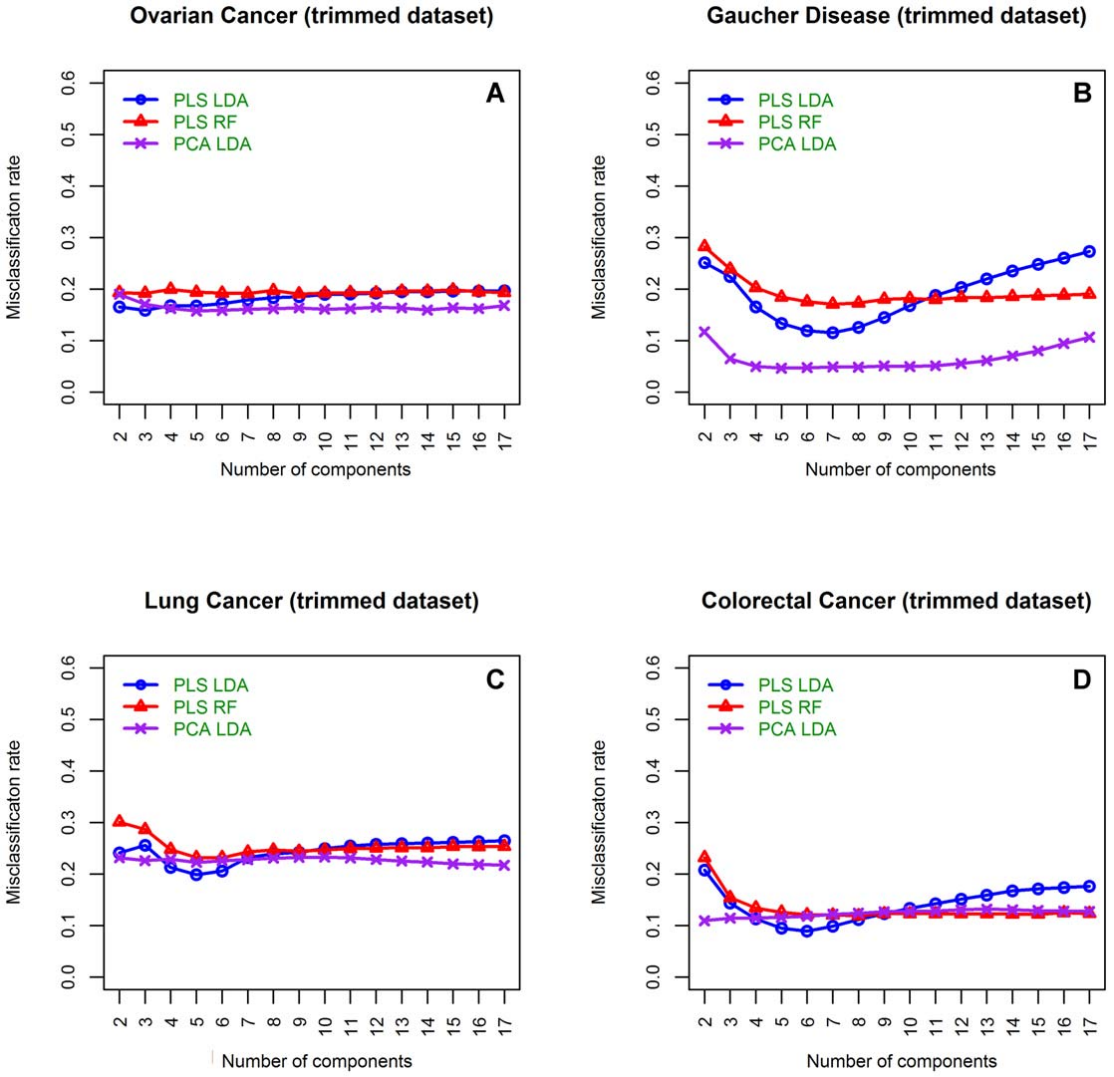
Misclassification rates of dimension reduction classifiers using the trimmed datasets. Mean misclassification rates for each of the dimension reduction-based methods using the trimmed dataset to build the classification model. **A**) Is from the OC dataset [16], **B**) is from the Gaucher disease dataset [46], **C**) is from the LC datasets and **D**) is from the CRC dataset [14]. Blue circles illustrate PLS-LDA classification results, red triangles are from a PLS-RF classifier and purple crosses show results obtained from a PCA-LDA classifier.

In terms of model parsimony, PLS-based methods performed well; in most cases these models utilise the least number of components and resulted in the lowest MCRs. With respect to the Gaucher data, a monotonic decrease across the number of components analysed in the PCA-LDA classifier was observed when using all variables (*Figure 1*). On this dataset, PCA-LDA resulted in the lowest MCR rate when all the variables were used. However, this was at a cost to model complexity as all 17 components were required to reach this accuracy. Furthermore, using the trimmed Guacher disease dataset, PCA-LDA outperformed both PLS-LDA and PLS-RF methods with lower MCR using a smaller number of components (*Figure 2*).

Both the LC and CRC datasets produced comparable results. More specifically, for the LC data the decrease in MCR for the PLS methods between variable selection and no variable selection was not followed by a decrease in model complexity, with the same number of components being suggested for both the full dataset and the trimmed set (*Table 1*). This is in sharp contrast to the PCA classification method which somewhat counter intuitively required considerably fewer components in the full dataset to get a MCR comparable to that in the trimmed dataset. Recall that these datasets have already been pre-processed to include variables that best discriminate between groups. Thus the performance of the PCA classification methods is not surprising as the optimal variables were present in the untrimmed data. The CRC results were almost identical to the LC dataset results. The only difference being that variable selection decreased the number of components in the PLS-LDA model from 6 to 5 which resulted in an identical MCR rate of 0.089. Therefore PLS-LDA demonstrated superior classification with a smaller number of variables than PLS-RF and PCA-LDA on both of these datasets.

The OC cancer dataset was by far the most complex set analysed here. Again, the PLS-based methods outperformed PCA-LDA when no variable pre-selection was employed (*Table 1*). However when variable pre-selection was performed the MCR was almost equal between PLS-LDA and PCA-LDA, although PCA-LDA did require an additional 2 components to reach equivalence with the PLS based method (3 components for PLS and 5 for PCA), see *Table 1*.

In order to gain insight on the differences between each of the dimension reduction techniques, and the effects of including additional components into the model, the MCRs for each classifier built on each of the untrimmed datasets is summarised in *Figure 1 A* through *D*. In every dataset the first five to seven components in the PLS-LDA method demonstrate the best classification rate. This observation is probably due to PLS's capacity to retain the important information in the earlier components when many mass units are used to build the model. More than seven components either increase or stabilise the MCR such that the addition of more than 7 components adds little value to the classification model (*Figure 1 A–D*). Of the dimension reduction methods tested, the PLS-RF approach performed most poorly. Specifically, while a decreasing trend in MCR was observed using fewer components, similar to the PLS-LDA method, the MCR was consistently higher than the PLS-LDA approach using the first seven components (*Figure 1 A–D*). However, the MCR stabilised using the PLS-RF method with the addition of PLS components to the model. Whereas, the MCR was not greatly improved by the addition of more than four components to the PCA based method (*Figure 1 A–D*).

Each classifier's accuracy was lower when variable selection was not performed compared to when the top 30 variables (trimmed dataset) were used (*Figure 2 A–D*). The exception to this was the LC and CRC datasets as they had undergone variable selection prior to this study which may explain why they performed better than the unmodified Gaucher and OC datasets. Again the PLS dimension reduction methods show most of the valuable variability within the data is retained using less than seven components, while, the addition of further components adds no value to the classification model. In addition, as observed for the full data set, the MCR stabilised when more than six components were used in the PLS-RF method (*Figure 2 A–D*).

### SVM classification

Unlike PCA and PLS, SVMs do not utilise a component space, as such the number of reduced dimensions does not need to be optimised. After deciding which kernel to employ, the only parameter that needed to be tuned was the *cost*, or *C*-term. On the untrimmed data, changing the *C*-term did not affect classification in the Gaucher and OC datasets with a MCR of 0.204 and 0.266, respectively. Whereas, a cost equal to 0.1 produced the lowest MCR in the untrimmed LC and CRC datasets. A *cost* of 0.1 also resulted in the lowest MCR using the trimmed data in all datasets (*Table 2*).

**Table 2:** SVM tuning results.

Dataset	Cost	Full dataset (MCR)	Trimmed dataset (MCR)
Gaucher	0.1	0.204	0.05
	1	0.204	0.052
	5	0.204	0.052
	10	0.204	0.052
CRC	0.1	0.102	0.112
	1	0.105	0.139
	5	0.105	0.156
	10	0.105	0.156
Lung	0.1	0.182	0.168
	1	0.183	0.192
	5	0.183	0.207
	10	0.183	0.208
Ovarian	0.1	0.266	0.15
	1	0.266	0.165
	5	0.266	0.166
	10	0.266	0.166

The performance summary (**MCR** = Misclassification rate) of a SVM-based classifier for both the full dataset (“full”) and the trimmed dataset (“trimmed”) that underwent variable selection using a univariate moderated t-statistic. These are mean values based on 1000 bootstrap samples for each dataset except for the OC data which used 200 bootstrap samples.

### Summary of all classifiers

**Figure 3:**
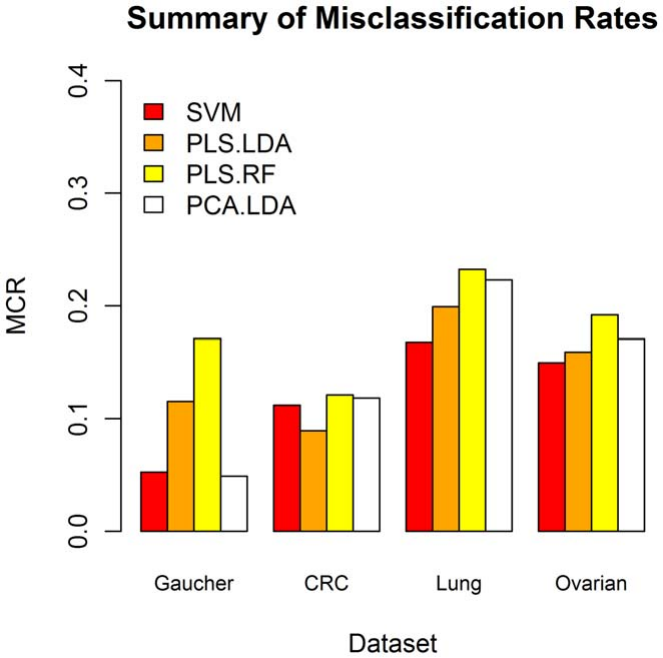
Summary of Misclassification results for all classifiers. Summary of mean MCR results for each of the optimised classifiers on each trimmed dataset. These results demonstrate the MCR for each classifier using the optimal number of reduced components from the PLS-LDA (excluding SVM). Gaucher data uses a 7 component model, the LC data uses a 5 component model, the CRC data uses a 6 component model and the OC data uses a 3 component model for each.

Based on the results, the SVM's were almost always the best classifier, except in the CRC dataset where PLS-LDA produced a MCR of 0.089 and in the Gaucher dataset where a six component PCA-LDA model produced a MCR of 0.046 compared to 0.112 and 0.05, respectively, using the SVM approach (*Table 1* *&* *2*). It is important to remember however, that the CRC dataset has been pre-filtered. In addition, when the same number of components are used to build the classification rule in both the PCA-LDA and PLS-RF methods as those used in the optimised PLS-LDA method (lowest PLS-LDA MCR) our data indicate that the PLS-LDA method resulted in a lower MCR than either PCA-LDA or PLS-RF methods in all cases but one (*Figure 3*).

A key advantage that the dimension reduction techniques have is that they yield loadings which represent feature-disease status associations in a computationally efficient manner. SVMs derive the decision boundary based on a small number of observations occupying the margin of the space contiguous between the groups. For this reason, any classification rule (and therefore individual feature loadings) derived from the support vectors will not have the theoretical underpinning afforded to the classification rules derived from PLS or PCA. In both PCA and PLS based methods, assumptions about linear associations among features, and a multivariate distribution of observations in the feature space (not an unrealistic assumption for this type of data) allow posterior probabilities to be calculated for individual observations. For this reason, the statistical PLS-based approaches offer some strong advantages over the machine leaning-based SVM procedure.

**Figure 4:**
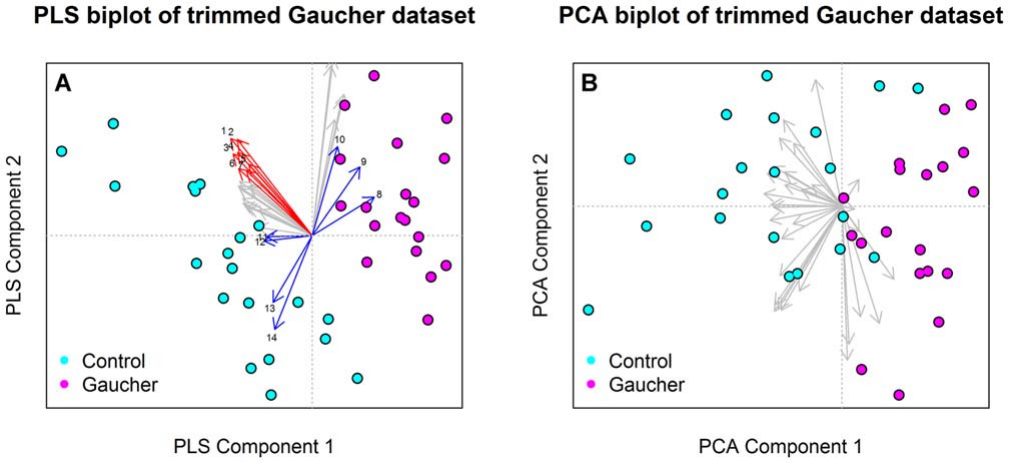
Comparison of PLS and PCA for dimension reduction. These plots demonstrate the capacity PLS has to separate classes based on the top 30 variables (*Figure 4A*) in the Gaucher dataset when compared to PCA (Note that this class separation is being heavily influenced by the loadings highlighted in Blue. Additionally, the vectors highlighted in red explain the within class variation in the control group. This is a key advantage PLS has over other methods.

Moreover, due to the supervised nature of PLS, it does a far better job of extracting between-class variation while demonstrating the variables that explain this variation compared to PCA. An example of this is in variables 7, 4, 5, and 10 in *blue* within *Figure 4 A* which seem to be influencing the separation between disease and control groups in the Gaucher dataset. Another valuable utility of PLS loadings are to display the within-class variation, for example the control group is spread out compared to the Gaucher group. This variability seems to be partially influenced by a cluster of mass units highlighted in *red*, see *Figure 4 A*. For comparative purposes a biplot using PCA on the same 30 variables is presented in *Figure 4 B*. From this it is clear to see the separation isn't as clear between each cohort, as such, it is not as apparent which variables are important in explaining differences between disease and control groups.

An additional disadvantage of the SVM procedure is it is only appropriate for two class problems. Although SVMs can be run on a pairwise basis for the classes >2 case, assessment of feature loadings and graphical representations are much less likely to be valid using a SVM strategy for the same reasons stated above. In contrast, the PLS approach can be formulated for any number of groups.

## Discussion

In this work we concentrated on the number of components that best performed classification of disease or non-disease groups under cross validation. In a clinical setting, readers are advised to calculate the optimal number of ***k*** *_PLS_* components based on some similar cross-validation criteria. In such canonical approaches, knowledge of disease state is required only for the learning set. After a suitable model has been trained, calibrated and validated using this learning data, estimates of disease class from unseen patients may be calculated without *a priori* knowledge of disease class.

The performance of the classification methods considered was highly variable across the datasets we used. Indeed, there was no single method that was convincingly superior across all of the datasets, suggesting that the final classifier to use should be based on a dataset by dataset basis after testing multiple classification rules. Additionally, if investigation into feature-disease status associations is of particular importance to the study design, perhaps a PLS based methodology should be adopted. We utilised PCA-LDA primarily due to its heavy use as a visualisation tool in ‘omics’ data [23]–[25]. PCA-LDA is not designed to capture between group variability and for this reason we do not advocate the use of PCA-LDA as a classification method. A challenge in this study was to meaningfully compare methods. Given the LC and CRC datasets had already undergone some degree of pre-processing (e.g. filtering) it was important that each of the datasets were reduced to the top 30 variables to meaningfully compare each method. While each of these methods is capable of handling more mass units than there are observations, filtering is generally recommended to remove the fraction of mass units that are not differentially present across classes [26], as well as to reduce the number of possible false positives. Another point of view is that the analytical approach should incorporate both feature selection and classification within the one model, this process is possible using a PLS-based method.

We have demonstrated here that PLS and SVM show strong utility for the generation of good classification results, even in the absence of dataset filtering. In fact, we believe using several methods for feature selection and classification may not be preferable to a single method (e.g. PLS) to both identify important masses and build a classification rule. PLS, unlike many filtering approaches (including the linear approaches used here) is a wrapping method as it formally takes into account the correlations among the mass units. A key pitfall of SVM is it's “one-to-all” approach [27] to a multiclass classification problem, unlike PLS-based classification which has been applied previously to the multiclass problem with promising results [28].

In this study design we employed a large number of learning sets in order to gain confidence in the accuracy of the MCR. The Gaucher dataset alone, produced by Surface enhanced laser desorption/ionisation – Time of flight/Mass spectrometry (SELDI – TOF/MS), responded the best using a combination of variable selection and PCA-LDA, based solely on MCR. This dataset aside, the general performance of PCA-LDA resulted in a loss of model parsimony which was the key disadvantage of this method across each dataset. Taking both parsimony and MCR into account, the PLS-LDA approach demonstrated more consistency across each of the datasets. As expected all approaches improved when variable pre-selection was implemented prior to dimension reduction and classification, except for the LC and CRC datasets which already contained the optimized variables and thus filtering only reduced the classifiers ability to distinguish case from control due to reducing the number of significant variables.

Here we compared a range of methods on a range of datasets produced using different Mass Spectrometry platforms. There are a number of possible approaches available to analyse multi-dimensional proteomic data. Currently, both machine learning and multivariate statistical methods are used within the microarray and proteomic fields with varying degrees of success. Machine learning methods have advantages of dealing with non-linear relationships but do not provide useful information for modelling variability in proteins. Alternately, multivariate statistical approaches are generally limited to linear associations but lend themselves to the explicit modelling of proteins or derived combinations thereof (i.e. latent components). In addition, it may be desirable to move towards methods where feature selection and classification are performed together in the one method. As such PLS offers great potential for the analysis of high dimensional proteomic data. In addition, further research on the capacity and utility of classification methods from proteomic classification should involve simulated data.

## Methods

### A proteomic dataset

A typical proteomic data matrix ***X*** *_ij_* will consist of response variables in the form of protein or peptide intensities and is composed of ***i*** rows (observations, participants), and ***j*** columns (proteins, peptides, m/z). Note that from here on the term “mass unit” will be used to represent proteins, peptides or mass over charge (m/z) units. In the case of classification, a vector of dummy variables, **y** *_i_* is coded to identify group membership of the observations. For multiclass cases, **y** *_i_* is extended to **Y** by constructing an indicator matrix.
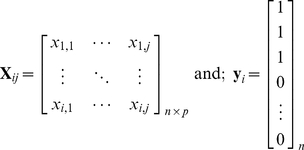
(1)


### SVM

Support Vector Machines (SVM) determine the optimal hyperplane between each class using only those training points which lay closest to the decision boundary. The points laying on the boundaries are so called “support vectors” and the space between is the margin. Support vectors from each class are maximised such that the centre of the margin becomes the optimal decision boundary (hyperplane). This is done by mapping *x_i_*∈<$>\scale 88%\raster="rg1"<$>ℝ*^d^* into a high dimensional feature space using a linear or nonlinear function 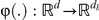. As discussed above, proteomic data typically contains a small number of observations with a large number of variables. Such conditions make it unlikely that classes are not linearly separable on the learningset, however this often results in a model that is overfit to the training data and not applicable to test data. Additionally, given the complexity (i.e. erroneous signal through noise etc.) and overlapping nature of classes in real-world data the expectation that each of the unseen test classes are linearly separable based on the learning model might be unrealistic. For this reason leniency of misclassified data points in and around the margin are tolerated by:
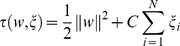
(2)Adjusting the regularization constant, *C*>0 affords a balance between classification accuracy and margin size. If *C* is too large, there is a high penalty for nonseparable points and as a result may store many support vectors and overfit. If it is too small, underfitting may occur. Here *w* represents an unknown vector with the same dimensions as ϕ(*x*).

*Equation 2* is solved using the Lagrange multipliers 0≤*α_i_*≤*C*, the solution of which is obtained by:
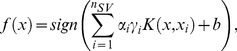
(3)where ***n*** *_SV_* represents the number of support vectors, *b*∈<$>\scale 85%\raster="rg1"<$>ℝ and *K(.,.)* is the kernel function which enables the representation of the training data in the feature space 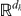 without ever leaving <$>\vskip -1\scale 85%\raster="rg1"<$>ℝ*^d^*. The kernel function can take many forms including: Linear, Gaussian, polynomial and radial basis function kernels [29], [30].

A key advantage SVMs have over other methods is their ability to manage both linear and non-linear classification problems, although their application to multi-class problems is limited due to their dependence on a one-to-one approach. This problem is often circumvented by representing a multiclass problem as several binary classification problems (i.e. one-to-all) [27]. For further details on SVMs, including a solid theoretical overview, see Burges (1998) [31] and for practical applications of SVMs see Luts, *et. al.* (2010) [27].

### Principal Components Analysis (PCA)

Principal components analysis is a popular dimension reduction method used to explore variation in complex datasets. The objective of principal components analysis is to summarise the data in as few dimensions as possible without losing an excessive amount of information. This is done by decomposing the data matrix, **X** *_ij_*, such that it is the product of a *scores* matrix **T** *_ik_* and a *loadings* matrix **P** *_ij_*. Note that ***k*** *_PCA_* represents the number of components or latent variables extracted from the data, such that each observation can be represented as a point in ***k*** *_PCA_*-dimensional space. This relationship is typically summarised by:
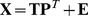
(4)where **E** denotes the residual error calculated from the deviations between the original values and their projection onto the new set of latent variables (components).

Prior to determining the latent variables, it's conventional to appropriately pre-process the original matrix of intensities first. Note that this is not related to the signal pre-processing required on particular types of MS data e.g. SELDI - TOF/MS or MALDI - MS1 outputs. Data pre-processing prior to PCA usually involves performing one of the following: 1) taking the covariance matrix and first centring the data then calculating the outer product (**XX** *^T^*); 2) using the correlation matrix which is a result of centring and reducing **X** to unit variance followed by calculating **XX** *^T^*; or 3) leaving the data un-centred and un-standardized to unit variance, the resulting **XX** *^T^* is the sums of squares and sums of cross-products matrix.

Centring the data involves displacing the origin such that the global mean vector is equal to zero, while reducing the data to unit variance allows all variables to contribute equally to how the observations are presented in the reduced dimensional space, irrespective of the individual variance of each. This is particularly useful if the magnitude of each mass unit's variance does not relate to its comparative importance. Thus if only centred (covariance matrix) data are entered into the PCA algorithm the effect of individual m/z's will have a greater influence on how the observations are seen in the lower dimensional space, while centering and scaling (correlation) reduces any effects due to m/z's with large variances. If the variables are all in the same units and are the same kind, the covariance matrix is often used. When implementing PCA it is important to note that different software packages use different pre-processing techniques.

In summary, PCA attempts to construct linear combinations of the original variables that are linearly independent (orthogonal) of each other. This is done in a way that attempts to preserve the euclidean distance among observations, that is, when the original observations are projected onto the new latent variables, the relative distance between objects in the original data and the new ***k*** *_PCA_*-dimensional space is conserved.

### Partial Least Squares (PLS)

Partial Least Squares is a canonical projection method which offers promising supervised dimension reduction capacity; this technique is used on datasets containing class membership variable/s, **y**, and predictor variable **X**. Unlike other popular dimension reduction techniques, such as principal components analysis, the PLS algorithm calculates each latent variable from **X** based on **y**. The objective is to maximize the covariance between **y** and **X**, unlike PCA which maximizes the variance of the variables, **X**, alone. Thus PLS, unlike PCA, explicitly accounts for the covariates (e.g. class membership) within the model.

In PLS, the latent variables (***k*** *_PLS_ = 1,…,p* where ***k*** *_PLS_*≤***k***) are a product of the iterative decomposition of **X** and **y** such that the original variables (mass unit intensities) get projected to a lower dimensional space where a sequence of bilinear models are fitted by ordinary least squares (at least originally this was the case), hence the name partial least squares [32]. This is especially true for the NIPALS method, however, later implementations use an eigen-analysis approach which brings it into line with most other classical multivariate methods. The goal of PLS is to find the linear relationship between the response and explanatory variables **y** and **X**:
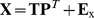
(5)

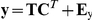
Where **T** represents the scores (latent variables) that our data have been projected down to, **P** and **C** are loadings and **E**_x_ and **E**_y_ are the residual matrices obtained from the original **X** and **y** variables.Determination of the lower dimensional components requires:
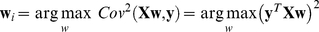
(6)Subject to 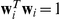 and 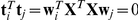, as described in [17].

The general PLS algorithm works as follows:

1. PLS components are calculated as the latent variable which maximizes the covariance between **X** and **y**;
2. The variance (information) from this component is removed from the original **X**-data, a process known as deflation. The remaining residual matrix has equal column and row lengths to the **X**-data, only the intrinsic dimensionality has been reduced by one; and
3. The next PLS component is calculated from the current residual matrix, and as in step 1, results in maximum covariance between **X**_(**1,…,***j*)_ and **y** subject to the constraint that it is mutually orthogonal with the previous one. This is repeated iteratively until little more improvement to the modelling of **y** can be achieved or **X** becomes a null matrix.

Note that deflation of the **X** matrix is carried out differently between the numerous PLS algorithms available i.e. NIPALS, SIMPLS and Kernal PLS algorithms [33], [34]. An overview and history of PLS may be found in Geladi and Kowalski (1986), Wegelin (2000), Martens (2001) and Wold (2001) [33], [35]–[37].

### Classification

While PCA and PLS can provide class separation on a qualitative level, they are not strictly classification methods themselves, but are dimension reduction techniques. Typically it is used in conjunction with existing classification methods.

#### Linear Discriminant Analysis (LDA)

In the context of this paper, LDA seeks to find a linear combination of the new components, **T**, obtained from the preceding PCA or PLS dimension reduction process. A key pitfall with the implementation of LDA is its inability to deal with a **n**≪**p** dataset. As such, incorporating PCA or PLS prior to classification will result in a reduced dimensionality of the original **X** data that is better handled by the formal model that LDA provides. This is done by projecting the observations onto this new co-ordinate system and passing them onto the classifier. A model is then developed to predict the class of an unknown observation based on prior probabilities calculated from a learning set, **L**_i_. Ideally these priors are maximized for group membership in order to get the best separability. The linear combination is calculated such that it maximizes the ratio of between-class variance relative to the within-class variance:
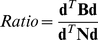
(7)Where **B** is the between class sample covariance matrix such that:
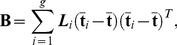
(8)and **N** is the within class sample covariance matrix,
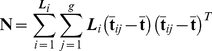
(9)
**d** is the direction that best distinguishes the difference between each class in the reduced dimensional space determined through PLS or PCA. Note that ***L*** *_i_* is the learning set observations. LDA assumes that the data are multivariate-normally distributed with equal variance/covariance matrices.

#### Random Forests

The Random Forests algorithm was formulated by Breiman (2001). Although a relatively new method, it has gained popularity for use on a wide range of linear and non-linear problems, partially due to being a model-free approach. Based on decision trees, a Random Forest is a classifier produced by aggregating individual tree predictors which have been built using ***i*** randomly sampled bootstrap observations from the original data [38]. Each tree in the forest is completely grown (i.e. no pruning) from each observation which are selected through bootstrap sampling. Trees are grown based on a decision criteria determined at each node; the number of trees can be selected based on the number of observations included. The combination of bagging and random variable selection to grow each tree produces a powerful tool with appealing characteristics for use on quantitative proteomic data. In addition, Random Forests can provide valuable information on variable importance, although research in this area is currently ongoing to reduce variable selection bias problems [39], [40]. When applied in conjunction with PLS the algorithm works as follows:

The RF algorithm builds an ensemble of classification trees which constitute the forest by:

1. For **T**_1,…,***k****PLS*_ latent variables a large number of random samples, **S**_1,…,*j*_, are obtained **z** times with replacement (bootstrap samples). Due to the nature of bootstrap sampling some observations may be observed once or may be present in replicates, while others will not be represented at all. Those that have not been selected are termed out-of-bag (OOB) data and need to be known. The random selection of predictors reduces the correlation between the trees in the forest.
2. For each node in each tree, **r**, input variables (vectors in our PLS-reduced dimensional space) are selected randomly as potential predictors on which the dataset is split. Unlike the samples in step 1, these are not bootstrap samples. The tree is then grown to completion with no pruning and the OOB data is used to estimate the error value of that tree.
3. Steps 1–2 are repeated, thus for each sample, **S_1,_**_***…,j***_, a classification tree is built resulting in a forest comprised of multiple trees.
4. An unknown sample is classified by running it through each of the trees in the forest where the resulting solution is produced by a weighted or unweighted majority vote [41]. It is the forest that constitutes the classification model.

Note that **S** and **r** are input by the user. It is suggested that **r** is set to √**p** [17] where **p** is the number of input variables which, in this context, is equal to the total number of variables in the matrix **T**.

RF is a popular method that has gained recognition for its ability to construct robust classifiers and select discriminant variables in proteomics [42], [43] and microarray fields [17], [39], [44]. A fundamental description of the method can be found in Malley *et. al.* (2011) [41] and for a more mathematical based description see the original works by Breiman (1996 and 2001) [38], [45].

### Datasets and data pre-processing

Each of the above techniques have been applied to several published proteomic datasets. Each dataset was chosen as they represent a number of diseases and popular Mass Spectrometric platforms. These include:

- A lung cancer (LC) and colorectal cancer (CRC) dataset which both contain 50 cancer cases along with 50 and 45, respectively, matched healthy controls. Both datasets have already undergone variable pre-selection and as such contain 39 (LC) and 109 (CRC) variables (mass units). They were both acquired using MALDI – TOF/MS technology and have undergone the appropriate pre processing steps as outlined in Schleif *et. al.* (2009) [14]. Their focus is on classification using a novel supervised relevance neural gas algorithm.
- An ovarian cancer (OC) dataset acquired via MALDI – TOF/MS with 47 cases and 44 controls and contains 24262 spectral features. The data set is available at http://bioinformatics.med.yale.edu/MSDATA/ and has already undergone the appropriate pre-processing steps outlined in Wu *et. al.* (2003) [16]. The authors investigated the utility of several classical methods of classification including LDA, Quadratic Discriminant Analysis (QDA), KNN, Aggregated classifiers, RF and SVM. In addition, they utilised variable importance measures from RF and the univariate t-statistic for variable selection creating a 15 and 25 variable dataset. Each of these reduced datasets were then analysed through the classification models. Additionally, they highlight the convergence issues when using LDA and QDA as standalone classifiers on high dimensional data.
- A Gaucher disease dataset consists of SELDI – TOF/MS spectra acquired from the serum of 20 Gaucher disease cases and 20 controls [46]. One of the cases has been removed as a potential outlier based on the authors recommendation [46]. This data contains 590 variables and the spectra have been pre-processed according to Smit *et. al.* (2007) [46]. The authors employed a PCA-LDA and validated its classification capacity with a permutation test followed by its predictive ability via a double cross-validation approach. They found a 15 component PCA-LDA model provided the strongest single cross-validation error.

### Study design

#### A note on data analysis

All processed data was analysed in the ‘*R*’ statistical computing and graphics program, version 2.11.1 (www.r-project.org), unless otherwise indicated. The *CMA* package was primarily used for most of the classifiers described in this manuscript [30]; while, our own PCA-LDA rule was developed and implemented within the *CMA* frame work. Each dataset contains a binary dummy variable set to either 0 or 1 indicating group class.

#### Comparison of different classifiers

Learning sets, **L**, and test sets, **T**, were created for each dataset via bootstrap sampling. Learning sets were built using randomly selected observations consisting of a fixed ratio equal to two thirds (0.66) of the original dataset, **S**. For each classification algorithm ***k***-bootstrap learning sets were created and aggregated, producing a learning matrix consisting of size 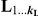. For the LC, CRC and Gaucher datasets ***k*** **_L_** was set to 1000. For the OC dataset ***k*** **_L_** = 200 bootstrap learning sets due to the large number of variables and computational intensity required by some of the classification methods.

Due to the range of different classifiers employed in this study, each classification rule generated and tested was assessed based on a global misclassification criteria generated from each of the test sets. That is, the number of times a classification rule created from 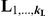 misclassifies a sample from the test set, **T**.

#### Comparing classification based on all variables to that based on preselected variables

From a biologist's perspective an important step, particularly within proteomic studies, is to later identify the panels of mass units with which the final classifier is built on. This is generally more important for techniques that don't acquire this additional data such as SELDI - TOF/MS and MALDI-MS1 methods. For example a biologist will often want to identify a particular mass in order to reveal its biological relevance which could then be used to inform future research directions. One view point is that the variables should be preselected (filtered), to this end it is important to preselect the variables that will be input into the classification method.

Variable pre-selection was performed using the Linear Models for Microarray Data, “*limma*” [47] method which uses a moderated t or F statistic to select significant masses. From each dataset, the top 30 variables (i.e. lowest p-values) were identified for each of the 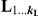 learningsets. Then a global top 30 were selected by counting and ranking each of the scores from the entire learningset matrix. These cross-validated global top 30 variables were then used for each of the trimmed models.

The effects of variable selection algorithms on classification error are not the primary theme of this manuscript, however, we include results with and without variable pre-selection for comparison.

#### Hyperparameter tuning

For comparative purposes each of the PLS-based methods was compared to two popular and well established algorithms currently used; one machine learning method (SVM) and one additional linear dimension reduction method (PCA-LDA). Both PLS and PCA contain one adjustable hyperparameter, **k**_PLS_ and **k**_PCA_ respectively, which is the number of components used to build the model. For all methods that involve dimension reduction i.e. the PCA-LDA, PLS-LDA and PLS-RF classifiers, a misclassification rate (MCR) was calculated using a 2–17 component model for comparative purposes. Not all dataset responded favourably when PCA or PLS models required greater than 17 components, so for reasons of comparison we limited dimensionality to 17. Caution is advised when deciding how many components to use; on the one hand too many components may increase classification accuracy, while at the same time over-parameterising the model may result in over-fitting. There is very little gained by setting the number of components too high as the model becomes superfluous with the additional parameters.

The SVM does not utilise a component space as is the case with the other methods. As such, prior parameter tuning of the SVM was performed and MCR was assessed between parameters. For each 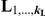 a SVM with linear kernel was tuned using four different “*C*” constants equal to 0.1, 1, 5, 10. The optimal parameter was selected based on the lowest MCR. All classifiers and subsequent analyses were performed on a SGI Altix 4700 (96 Itanium2 p9000 cores, 198 Gigabytes shared memory and a SUSE Linux Operating System).
